# Reviews

**Published:** 1888-01

**Authors:** 


### REVIEWS.

THE EQUINE DISEASES OF INDIA. By Richard W. Burke, M. R. C. V. S.; Jubbulpore, 1887.

This attractive book deals with the five most serious diseases of the Equines in India, and offers to the American reader some valuable points in etiology, symptoms and pathology in the same or analogous diseases, as they appear in the more Southern States and in Summer-heat. In "Surra" we find Mr. Burke's experience agrees with our own in failing always to associate parasites in the ætiology of Progressive Pernicious Anæmia. He has, however, misunderstood the "Scalma" of Prof. Dieckerhoff in considering it an analogous disease.

It would be interesting if one, with the experience of the author in Anthrax attacking different species of animals side by side, would explain if the variation of food and manner of feeding have any influence in determining the form of Anthrax as it infects the camel, horse or other cattle. In the essential form of "Kamri" we find ætiological analogy with our late summer endemics of the so-called "cerebro-spinal meningitis."

The article on "Barsati" has appeared too recently in the JOURNAL to require comment. R. S. H.

A MANUAL OF COMPARATIVE ANATOMY OF THE DOMESTICATED QUADRUPEDS. By Dr. Hormasji Edalji Sukhia, L. M. and S. Bombay. Anglo-Jewish and Vernacular Press, 1887, pp. 262.

We have perused this little volume with a great deal of interest, not alone on account of its intrinsic value, but also because of its publication in a land, whose mixed Hindoo and Mohammedan populations, entertain the most fanatical prejudices against the dissection of two of the most important animal species, with which the work deals. The latter, is not as the title would imply a complete manual of comparative anatomy, but deals merely with the skeleton, ligaments, and muscles. The visceral, neural and circulatory systems are evidently left for consideration in a second volume, although strangely enough this is nowhere explicitly stated.

As far as the work goes it is very accurate and complete. Valuable comparative tables of the muscles giving their synonyms and points of origin and insertion abound in the book. We have failed to detect a single material error, and the typographical ones are corrected by the author himself. So far India is ahead, and Mr. Sukhia deserves great credit for his painstaking and faithful performance of the task he has set himself. The book will not alone prove valuable to his countrymen, but it will materially lighten the work of junior students in the dissecting room. An excellent index facilitates ready reference. We are surprised that the elephant is not referred to, but this will prove no drawback in other lands than his own. ***

A POPULAR ZOOLOGY. By J. Dorman Steele, Ph. D., author of a series in the Natural Sciences, and J. W. P. Jenks, A. M., Professor of Agricultural Zoology in Brown University. A. S. Barnes & Co., New York, 1887.

On looking over this handy little volume we confess to having experienced a momentary envy, that in our young days we had not the advantage of similar royal roads to learning. In admirably selected English, convenient classifications and happy illustrations the essentials of Zoology can be almost unconsciously assimilated by a boy twelve to fourteen years old from this book. The wood cuts are numerous and well executed, some, particularly those of the mammalia are among the finest specimens of American xylography. Undoubtedly this practical readable and useful treatise will pass through a rapid series of successive editions. In that event we would suggest to the surviving author, that instead of giving only the inflection of the Latin terms used to designate genera and species, he give also their etymological derivation. When the pupil is told that pterodactylus, stands for wing fingered, he has killed a zoological and etymological bird with one stone. ***

L'Indirizzo e il Metodo nell inseguamento della Anatomia Veterinaria. Dal Dotter Lanzillotti-Bounsanti, Milano, 1887.

The author devotes thirty-two pages to the subject of Veterinary Anatomy, its history and the best methods of study. Alluding to its importance he says. "The notable progress lately made in Comparative Anatomy, has greatly extended the boundaries of Human Anatomy, raising it to a true scientific basis. Once established that the organization of man in its fundamental and essential features correspond to that of the other vertebrates, it became necessary to give to its study a new direction, rational and scientific. To accomplish this the study of Human Anatomy was not sufficient and the study of the lower animals became indispensable."

Practical Urine Testing. By Charles Godwin Jennings, M. D. Detroit: D. O. Haynes & Co., 1887.

This is a very small book but contains very important matter.

It tells in a clear and concise way how to analyse accurately the urine in health and disease.

Qualitative and quantitative tests receive particular attention and a chapter is devoted to microscopical examination.

The Pennsylvania State College Agricultural Experiment Station, H. P. Armsby, Ph. D., Director. Bulletin No. 1, October, 1887.

In the establishment of this station the object has been the solution of the many problems which have presented themselves to the attention of observing farmers who lack opportunities to solve them to their satisfaction.

Stock raisers will be especially interested in this movement, as it will afford them practical aid in determining questions as to the comparative value of different foods in fattening animals, the yield of milk of various cows under different circumstances, the quality of the meat and the relative proportion of fat and lean according as different methods of feeding are employed.

THE PHYSICIANS' VISITING LIST. P. Blakeston, Son & Co., Philadelphia, 1888.

This handy book is now in its thirty-seventh year of publication and this year comes out with several improvements, the many years experience of the publishers have enabled them to put this together in the best manner.

